# Massive parallel sequencing of mRNA in identification of unannotated salinity stress-inducible transcripts in rice (*Oryza sativa *L.)

**DOI:** 10.1186/1471-2164-11-683

**Published:** 2010-12-02

**Authors:** Hiroshi Mizuno, Yoshihiro Kawahara, Hiroaki Sakai, Hiroyuki Kanamori, Hironobu Wakimoto, Harumi Yamagata, Youko Oono, Jianzhong Wu, Hiroshi Ikawa, Takeshi Itoh, Takashi Matsumoto

**Affiliations:** 1 National Institute of Agrobiological Sciences (NIAS), Division of Genome and Biodiversity Research, 1-2, Kannondai 2-chome, Tsukuba, Ibaraki 305-8602, Japan; 2 Institute of the Society for Techno-innovation of Agriculture, Forestry and Fisheries, 446-1, Ippaizuka, Kamiyokoba, Tsukuba, Ibaraki 305-0854, Japan; 3 Hitachi Government & Public Corporation System Engineering Ltd., Solution Division 4, Research & Development Center Bioinformatics Group, 2-4-18 Toyo, Koto-ku, Tokyo 135-8633, Japan

## Abstract

**Background:**

Microarray technology is limited to monitoring the expression of previously annotated genes that have corresponding probes on the array. Computationally annotated genes have not fully been validated, because ESTs and full-length cDNAs cannot cover entire transcribed regions. Here, mRNA-Seq (an Illumina cDNA sequencing application) was used to monitor whole mRNAs of salinity stress-treated rice tissues.

**Results:**

Thirty-six-base-pair reads from whole mRNAs were mapped to the rice genomic sequence: 72.0% to 75.2% were mapped uniquely to the genome, and 5.0% to 5.7% bridged exons. From the piling up of short reads mapped on the genome, a series of programs (Bowtie, TopHat, and Cufflinks) comprehensively predicted 51,301 (shoot) and 54,491 (root) transcripts, including 2,795 (shoot) and 3,082 (root) currently unannotated in the Rice Annotation Project database. Of these unannotated transcripts, 995 (shoot) and 1,052 (root) had ORFs similar to those encoding the amino acid sequences of functional proteins in a BLASTX search against UniProt and RefSeq databases. Among the unannotated genes, 213 (shoot) and 436 (root) were differentially expressed in response to salinity stress. Sequence-based and array-based measurements of the expression ratios of previously annotated genes were highly correlated.

**Conclusion:**

Unannotated transcripts were identified on the basis of the piling up of mapped reads derived from mRNAs in rice. Some of these unannotated transcripts encoding putative functional proteins were expressed differentially in response to salinity stress.

## Background

Gene expression profiling is accelerating our progress toward a comprehensive understanding of the genetic mechanisms that control responses to environmental stress. Microarray analysis was developed to obtain overall gene expression profiles in various plants. Microarray profiling and the recently introduced tag-based sequencing approaches are proven technologies for estimating gene expression. However, array-based technologies have critical limitations [1,2]. As most microarray probes are designed on the basis of gene annotation, arrays are limited to the analysis of transcripts from previously annotated genes of a sequenced accession of a species. Probes are designed to cover only a very small portion of a gene and so do not represent the whole structure of the gene. Moreover, computationally annotated genes have not fully been validated, because ESTs and full-length cDNAs (FL-cDNAs) cannot cover entire transcribed regions. It is therefore important to identify whole transcripts (including unannotated transcripts) for complete gene expression profiling. There is a need for the development of technologies beyond arrays.

Sequencing-based approaches could overcome the limitations of array-based technologies. Following the rapid progress of massive parallel sequencing technology, whole mRNA sequencing has been used for gene expression profiling [3-8]. This sequencing involves mapping of the reads on known annotated gene models but cannot be used to identify novel genes. Recently, a series of programs have been developed for building gene models directly from the piling up of short reads: Bowtie efficiently maps short reads on genomic sequences [9]; TopHat concatenates adjacent exons and identifies reads that bridge exon junctions [10]; and Cufflinks [11] constructs gene models from the exons and bridging sequences predicted by Bowtie and TopHat and then calculates their abundances of these sequences. The use of this series of programs has the potential to discover new transcripts from mRNA-Seq (an Illumina cDNA sequencing application) but has only just begun [7,12].

In this study, we identified unannotated transcripts in rice on the basis of the piling up of mapped reads. As a model case, we give examples of salinity stress-inducible unannotated transcripts encoding putative functional proteins. For these purposes, we performed whole mRNA sequencing by using massive parallel sequencing technology. We also took advantage of various high-quality genomic resources in rice, including the genomic sequence (International Rice Genome Sequencing Project [IRGSP] build 4.0), FL-cDNA sequences [13], the Rice Annotation Project database (RAP-DB: http://rapdb.dna.affrc.go.jp/) [14,15], and a rice 44K microarray (Agilent Technologies, Palo Alto, CA, USA), in our analysis of rice transcriptomes. First, to estimate the scale of the transcriptomes in rice, we mapped 36-base-pair (bp) reads from the mRNA of salinity stress-treated rice tissues on the rice genome. The coverage of previously annotated regions or of the rice genome was then calculated. Second, we attempted to identify salinity stress-inducible genes as a model system for gene expression profiling by mRNA-Seq. The number of mapped reads was counted and marked on the rice genome. Third, using the mRNA-Seq data, we used Bowtie, TopHat, and Cufflinks to construct gene models based on the piling up of short reads on the rice genome, and compared these with previous annotations and then characterized the unannotated transcripts. We conducted a BLASTX search for the unannotated transcripts, and we discuss the function of the encoded proteins. Fourth, to validate our sequence-based technology, we compared the results of quantification by the array-based and sequence-based approaches, and we discuss the advantages of the latter. This work contributes to the discovery of whole salinity stress-inducible transcripts without the need to rely on previous annotations. It should help to establish further sequence-based gene expression profiling in any organism.

## Results

### Mapping of 36-bp reads to the rice genome

**Figure 1:**
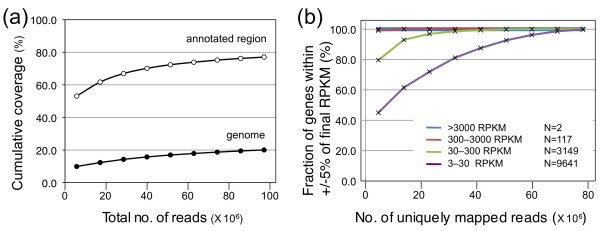
**Accumulation of 36-bp reads to cover whole transcripts**. (a) Cumulative coverage of rice genome and annotated region. Data from nine technical replicates of reads from roots after salinity stress were accumulated. Cumulative coverage was calculated by using reads uniquely mapped on the rice genome (black) or the RAP2 annotated region (white). As the number of reads increased, the cumulative coverage approached a plateau. (b) Robustness of the measurement of transcripts in four different expression classes. Saturation of sequencing was estimated on the basis of the fraction of RAP2 genes supported by FL-cDNA sequences that had reached their final RPKM (reads per kilobase of exon model per million mapped reads) [16]. Vertical axis indicates the fraction of genes for which the RPKM was within 5% of the final value, and horizontal axis indicates the cumulative number of uniquely mapped reads. The fraction of highly expressed genes was almost unchanged, whereas those of genes with relatively low expression converged slowly. *N *indicates the number of transcripts in each of the four classes.

We performed rice transcriptome analysis at single-nucleotide resolution by using Illumina mRNA-Seq technology. Briefly, poly(A) RNAs from salinity stress-treated rice tissues were reverse-transcribed and sequenced (Table 1). Millions of 36-bp reads were mapped to the rice genomic sequence (IRGSP Build 4.0), with at most two mismatches or 3 bp of indels allowed. To obtain many kinds of transcripts, data on nine technical replicates of the sequencing run of cDNA from the roots after salinity stress were accumulated. As the number of reads increased, the cumulative coverage of both the genome and the annotated transcribed region gradually approached a plateau (Figure 1a). Saturation of sequencing was also estimated on the basis of the fraction of genes that had reached their final RPKM (reads per kilobase of exon model per million mapped reads) [16]. As the number of reads increased, the fraction of highly expressed genes (RPKM ≥ 300) close to their final RPKM was almost unchanged, whereas those of genes with relatively low expression (RPKM 3-30) converged more slowly (Figure 1b). With four technical replicates (corresponding to about 27 to 35 million reads), 81.2% of genes with relatively low expression levels (RPKM 3-30) reached to within ± 5% of their final RPKM (Figure 1b). Thus, for further analysis, we adopted the summing of four technical replicates after filtration according to their base quality.

**Table 1:** Numbers of mapped reads

Sample	Total filtered reads	Unique						Multiple	%	Unmapped	%
		**total**	**%**	**genome**	**%**	**bridged**	**%**				

shoot_0 h	35,026,580	27,570,633	78.7	25,691,385	73.3	1,879,248	5.4	3,918,724	11.2	3,528,570	10.1
root_0 h	26,993,353	20,765,422	76.9	19,422,807	72.0	1,342,615	5.0	1,712,639	6.3	4,506,648	16.7
shoot_1 h	32,535,506	25,523,252	78.4	23,824,180	73.2	1,699,072	5.2	3,555,750	10.9	3,448,393	10.6
root_1 h	32,952,067	26,672,186	80.9	24,783,243	75.2	1,888,943	5.7	1,967,331	6.0	4,301,387	13.1

RNAs were prepared from normal shoot (shoot_0 h), normal root (root_0 h), shoot with salinity stress (shoot_1 h), or root with salinity stress (root_1 h). Numbers of 36-bp reads and their percentages of the total number of filtered reads, obtained by the summing of four technical replicates (Total filtered reads), are shown, as are reads mapped to the rice genome (Unique-genome), reads mapped uniquely to a predicted exon-exon bridging sequence (Unique-bridged), the total number of reads mapped uniquely to the genome and to a predicted exon-exon bridging sequence (Unique-total), reads mapped to multiple loci of the rice genome (Multiple), and reads unable to be mapped to the rice genome (Unmapped).

Rice transcriptome analysis was based on response to salinity stress. mRNAs were prepared from the tissues of normal rice shoots and roots and from those subjected to 1 h of salinity stress. Of the 27 to 35 million quality-evaluated reads (Table 1; Total filtered reads), 72.0% to 75.2% were mapped uniquely to the rice genome (Table 1; Unique-genome); 5.0% to 5.7% of the reads bridged flanking exons (Table 1; Unique-bridged); 6.0% to 11.2% of the reads were repetitive sequences (Table 1; Multiple); and 10.1% to 16.7% had no match in the genome (Table 1; Unmapped). Thus, a total of 76.9% to 80.9% of the reads were mapped uniquely to the rice genome or to exon-exon junctions (Table 1; Unique-total).

Of the unmapped reads, 26.1% had high levels of identity to sequences derived from sequencing adaptors (11.0%), contaminating organisms (8.2%), or ribosomal RNA (6.9%) (Additional file 1. Table S1). A few transcripts might have been transcribed from unsequenced genomic regions of rice [17]. However, most of the unmapped reads (71.5%) had no similarity to each other (data not shown). Our preliminary experiment showed that the ratio of these unmapped reads was higher with mRNA-Seq (10.1%-16.7%; Table 1; Unmapped) than with genomic sequencing (2.0%-3.1%; data not shown). Thus, part of the random sequences might have come from residual random primers used in cDNA synthesis. The common random sequences might have come from sequencing errors in the use of the Illumina sequencing technology.

### Identification of differentially expressed genes by mRNA-Seq

mRNA-Seq quantifies the amount of transcripts on the basis of the number of sequence reads mapped on each gene. We adopted this method for transcript quantification by RPKM [16] and calculated the RPKM of each gene (Additional file 2: Table S2). RPKM quantification was distributed from 0 to over 10^4^. In shoots under normal conditions, the gene encoding ribulose bisphosphate carboxylase activase (AK104332) was expressed at extremely high levels (rpkm_0 hr_shoot = 10612.237). In roots under normal conditions, the gene for metallothionein (AK105219) was expressed at extremely high levels (rpkm_0 hr_root = 23661.149). The statistical mean and median were 19.78 and 3.399, respectively, in the shoot, and 18.705 and 4.241 in the root under normal conditions.

**Figure 2:**
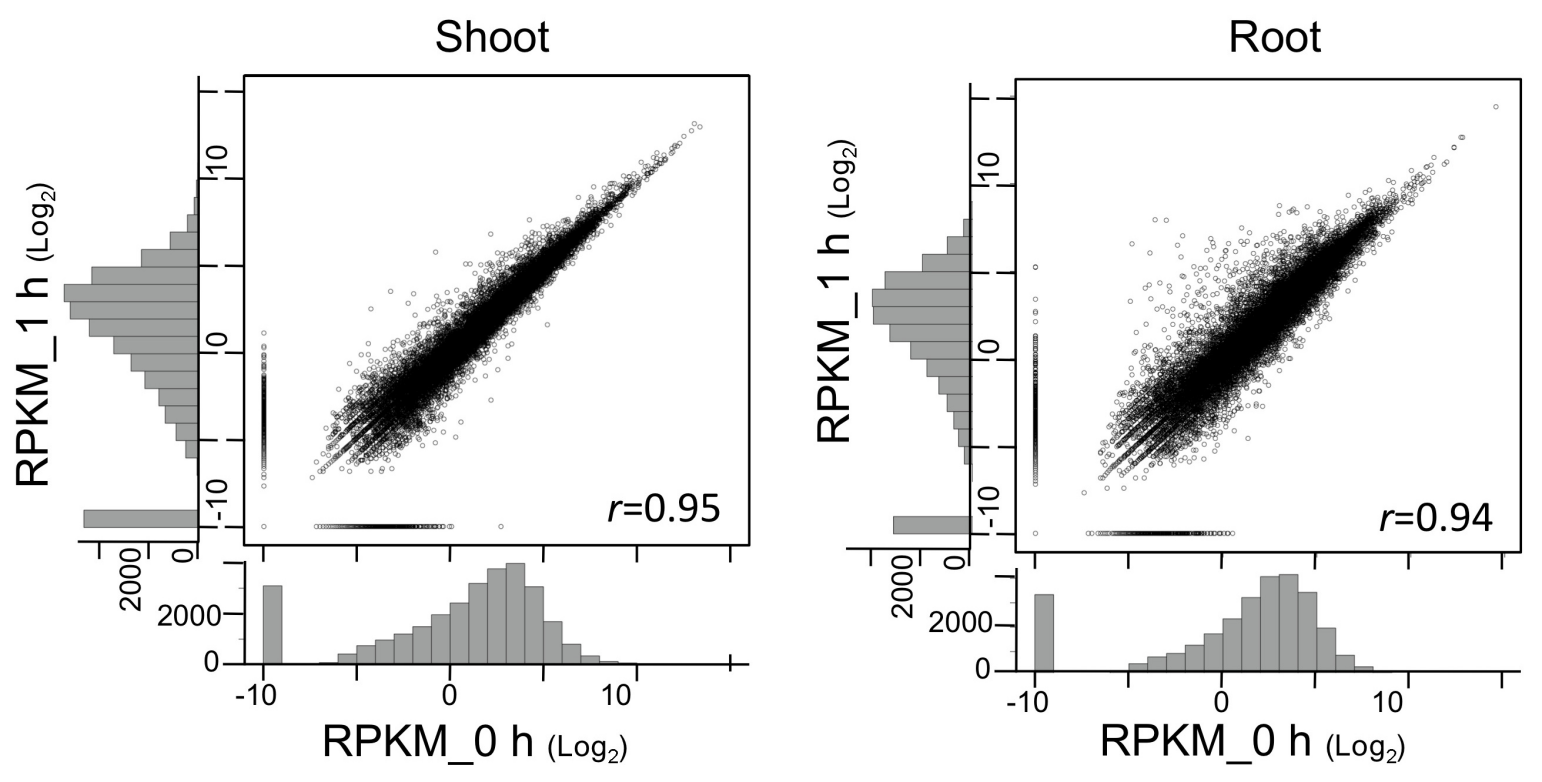
**Comparison of RPKM of each gene after salinity stress**. RPKM values for 29,389 RAP2 representative transcripts in the presence or absence of salinity stress were compared in the shoot (left) and root (right). For each gene, the RPKM (log_2_) value without salinity stress is plotted on the horizontal axis, and the corresponding RPKM (log_2_) value with stress is plotted on the vertical axis. Distributions of the number of transcripts are given outside the plot. The number of highly differentially expressed genes was greater in the root than in the shoot. Pearson's correlation coefficient is given in the corner of each plot.

We then comprehensively compared the RPKM of each gene in response to salinity stress (*r *= 0.95 in shoot and 0.94 in root; Figure 2). We used the G-test with a 1% false discovery rate (FDR) and identified 6,469 (in shoot) and 10,321 (in root) differentially expressed RAP2 genes. Of these, 3,050 (up, 1,651; down, 1,399) genes were commonly differentially expressed. The number of highly differentially expressed genes (> 32×), such as those encoding bHLH-containing protein (AB040744) and amino acid transporter (J075191I06), was greater in the root (58 genes) than in the shoot (5 genes). Expression of genes previously identified under salinity stress [18]--i.e. *OsTPP1 *(AK103391), *LIP9 *(AY587109), *OsABA2 *(AK062655), *OsMST3 *(AK069202), *WSI76 *(AK107065), and *MYBS3 *(AK107134)--was induced in the root (> 2×). For a complete comparison see Additional file 2: Table S2.

**Figure 3:**
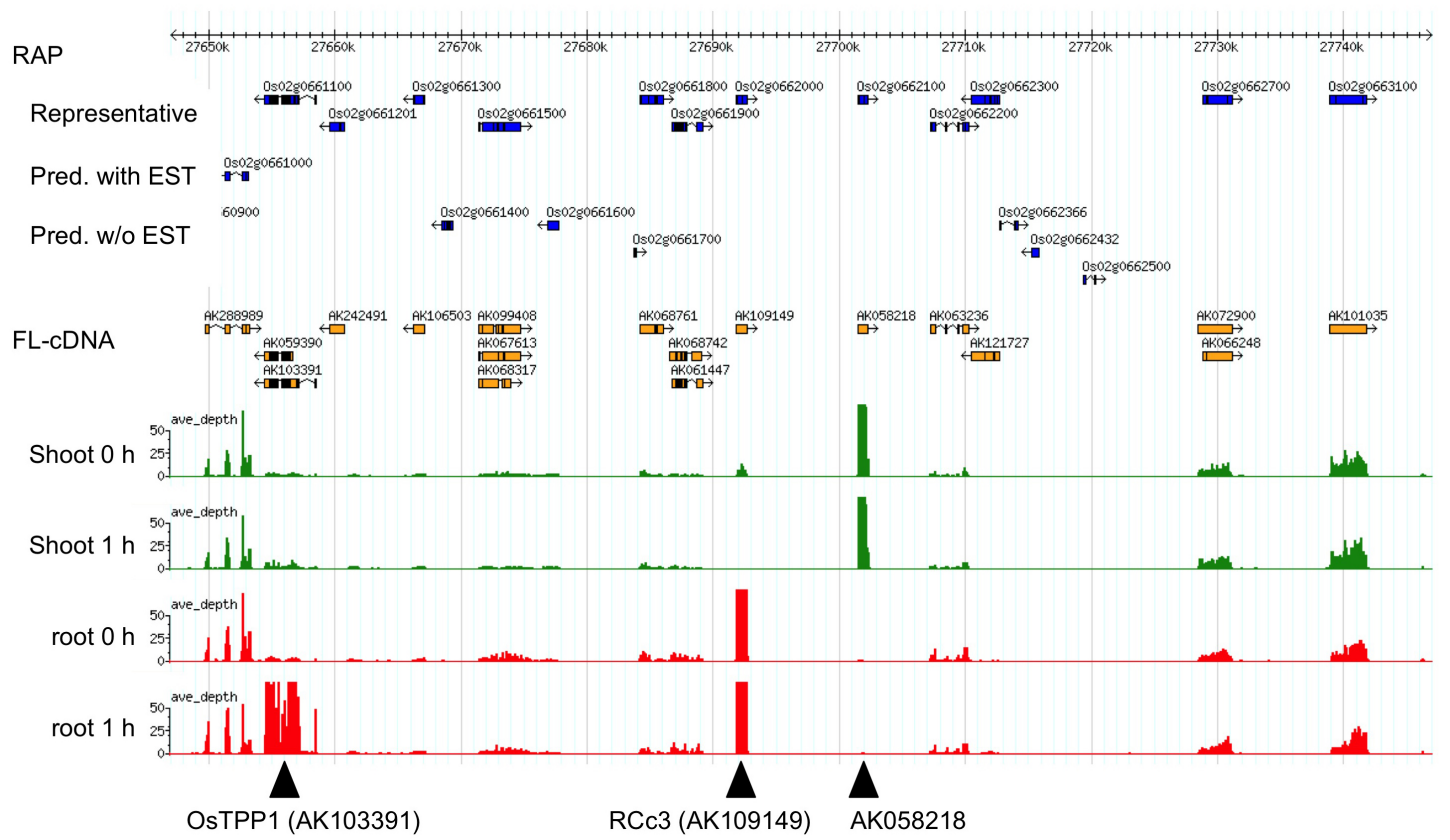
**Differential expression of genes in a 100-kb region on chromosome 2**. Gene models based on RAP representative loci (Representative), RAP predicted genes supported by ESTs (Pred. with EST) or not supported by ESTs (Pred. w/o EST), and full length-cDNA sequences (FL-cDNA) are shown. Graphs indicate the average depths of 36-bp reads on the rice genome (IRGSP build 4) by xyplot in GBrowse; each position represents the number of reads covering each 100 bp per million mapped reads. The level of expression is normalized against shoot 0 h (no saline exposure) as the standard. The genes *OsTPP1 *(AK103391), *RCc3 *(AK109149), and AK058218 were expressed differentially.

The distribution of mapped reads on the rice genome was graphed on a GBrowse [19] (Figure 3). For example, the *OsTPP1 *(for trehalose-6-phosphate phosphatase: TPP) gene (AK103391), which encodes a protein that synthesizes the abiotic stress-protectant trehalose [20,21], was expressed exclusively in the root after 1 h of salinity stress; *RCc3 *(AK109149), which was previously identified as a root-specific gene [22], was expressed only in the root with and without stress; AK058218 (similar to *ZmGR1a *in *Zea mays*) was expressed exclusively in the shoot; most of the neighboring genes were expressed evenly in all tissues used (Figure 3).

### Constructing gene models by mRNA-seq

**Figure 4:**
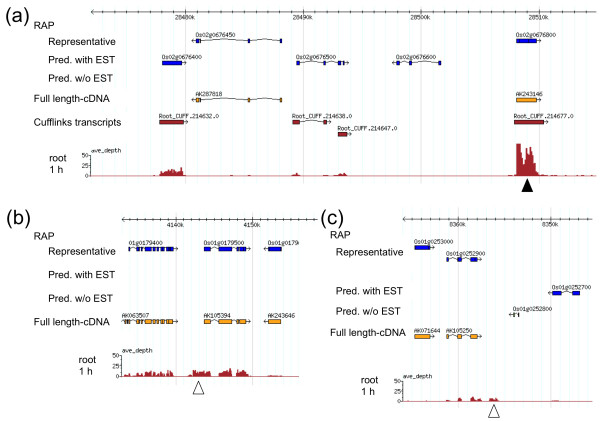
**Prediction of transcripts**. (a) Transcript prediction by Cufflinks program. Graph indicates the average depth of reads from mRNA-Seq after salinity stress of the root, as in Figure 3. Exon models (Cufflinks transcripts) were predicted by the piling up of reads (graph) by the Cufflinks program. RAP representative loci (Representative), RAP predicted genes supported by ESTs (Pred. with EST) or not supported by ESTs (Pred. w/o EST), and full length-cDNA sequences (FL-cDNA) are shown. AK243146, which is similar to *DREB1B *in *Arabidopsis thaliana*, was expressed and also predicted by the Cufflinks program (black triangle). (b), (c) Putative extension of RAP2 annotated transcripts. Putative transcripts might have a much longer 5' exon (b; white triangle) or 3' UTR (c; white triangle) than the annotations in RAP-DB. Full-length-cDNAs did not cover the entire transcribed regions (white triangles).

Transcribed regions were identified on the basis of the piling up of mapped short reads through the programs Bowtie [9], TopHat [10], and Cufflinks [11]. In the shoot, 51,301 transcripts were predicted (RPKM ≥ 2, length ≥ 100 bp) (Table 2); 94.6% (48,506/51,301) of the predicted transcripts were mapped on previously annotated loci in RAP2 [14,15]; thus, the remaining 2,795 predicted transcripts were unannotated in RAP-DB (Table 2). In the root, 3,082 of the 54,491 predicted transcripts were mapped on unannotated regions (Table 2). For example, the previously annotated gene AK243146, which is similar to *DREB1B *in *Arabidopsis thaliana *(GI: 3738226), was expressed after salinity stress and also predicted by Cufflinks (Root_CUFF. 214677.0); other exons were also predicted and connected by bridging sequences elucidated by TopHat (Root_CUFF. 214638.0) (Figure 4a). Reads were also mapped on the extended parts of the ends of most 5' and 3' exons in previous gene models (Figure 4b, c). Of the transcripts mapped on previously annotated loci, 1,738 (shoot) and 2,297 (root) had not been supported by ESTs [23] or FL-cDNAs [13].

**Table 2:** Numbers and ORF predictions of transcripts

		shoot	root
No. of transcripts mapped on RAP2 annotated loci		48,506	51,409
No. of transcripts mapped on unannotated region		2,795	3,082
	--ORFs detected by BLASTX	(995)	(1,052)
	--ORFs ≥ 20 a.a., no BLASTX hits	(1,670)	(1,873)
	--no ORFs or ORFs < 20 a.a.	(130)	(157)

Total		51,301	54,491

Transcripts were predicted on the basis of the piling up of mapped reads by the Cufflinks program (RPKM ≥ 2, length ≥ 100 bp). ORF prediction was conducted by BLASTX search against UniProt (Swiss-Prot) and RefSeq (reviewed and validated). ORF: open reading frame; a.a: amino acids.

**Figure 5:**
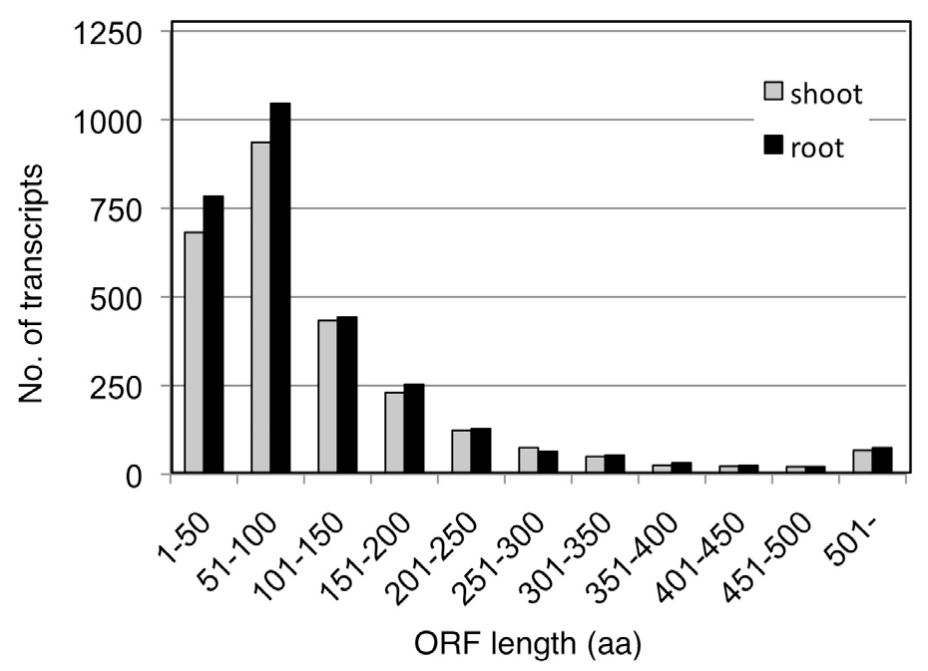
**ORF length and number of transcripts**. The distribution of ORF lengths of unannotated transcripts predicted by Cufflinks is shown. Vertical axis indicates the number of transcripts from the shoot (grey) and root (black), and horizontal axis indicates the ORF length of each predicted transcript.

We attempted to predict the functions of unannotated transcripts by BLASTX search and longest-ORF search. In a BLASTX search against the UniProt and RefSeq sequences, of the predicted transcripts, 995 (shoot) and 1,052 (root) had ORFs similar to those encoding the amino acid sequences of functional proteins (Table 2). Of the remaining unannotated transcripts, 1,670 (shoot) and 1,873 (root) had ORFs encoding at least 20 amino acids by longest-ORF search (Table 2). Amino acid length was widely distributed: the mean and median were 125 and 77 amino acids in the shoot, and 123 and 74 in the root (Figure 5). We used the G-test with a 1% FDR and identified 213 (up, 86; down, 127; in shoot) and 436 (up, 146; down, 290; in root) differentially expressed Cufflinks transcripts. Even though the lengths of Cufflinks transcripts were not completely identical between shoot and root, at least 55 differentially expressed transcripts were common to the two tissues. In response to salinity stress, 5 (shoot) and 13 (root) unannotated transcripts were upregulated (≥2×) (Table 3). These unannotated transcripts encoded, for example, proteins similar to indole-3-glycerol phosphate lyase and gibberellin 2-beta-dioxygenase (Table 3). Of the other differentially expressed genes (< 2×), Root_CUFF.256193.0 was upregulated (1.9×); it encoded proteins similar to MSL2 (MscS-LIKE2) (Additional file 3: Table S3). For a complete list of unannotated transcripts see Additional file 3: Table S3.

**Table 3:** Examples of expression ratios and putative functions of unannotated transcripts

shoot						
Cufflinks_ID	NT_Length	RPKM_0	RPKM_1	Ratio	AA_Length	Description

Shoot_CUFF.273129.0	146	1.333	13.8	10.4	48	Indole-3-glycerol phosphate lyase
Shoot_CUFF.412372.0	372	3.871	14.33	3.7	122	Inter-alpha-trypsin inhibitor heavy chain-related
Shoot_CUFF.436239.0	1,376	2.744	7.23	2.6	320	Pleiotropic drug resistance protein 4
Shoot_CUFF.379980.0	692	2.137	5.398	2.5	118	HHP2 (heptahelical transmembrane protein2)
Shoot_CUFF.81865.0	2,068	17.504	37.286	2.1	591	Alcohol oxidase-related

root						
Cufflinks_ID	NT_Length	RPKM_0	RPKM_1	Ratio	AA_Length	Description

Root_CUFF.327296.0	965	0	24.461	-	283	Gibberellin 2-beta-dioxygenase
Root_CUFF.298860.0	638	4.519	67.671	15.0	137	Putative lipoxygenase 5
Root_CUFF.439679.0	691	6.259	23.299	3.7	229	Probable pleiotropic drug resistance protein 1
Root_CUFF.168009.0	1,381	6.562	22.293	3.4	75	Phosphoinositide-specific phospholipase C
Root_CUFF.439685.0	1,382	3.8	12.555	3.3	336	Pleiotropic drug resistance protein 4
Root_CUFF.300686.0	473	1.415	4.607	3.3	157	Kinesin motor protein-related
Root_CUFF.177468.0	641	2.651	7.869	3.0	40	Phosphatidate cytidylyltransferase family protein
Root_CUFF.220154.0	636	1.7	4.695	2.8	212	Chromosome-associated kinesin, putative
Root_CUFF.365224.0	338	2.133	5.491	2.6	68	Binding/protein transporter
Root_CUFF.415910.0	372	6.228	14.968	2.4	122	Inter-alpha-trypsin inhibitor heavy chain-related
Root_CUFF.406739.0	669	1.462	3.317	2.3	48	VAD1 (vascular associated death1)
Root_CUFF.44438.0	639	2.256	4.736	2.1	118	Jacalin lectin family protein
Root_CUFF.450445.0	907	1.419	2.892	2.0	302	PDR11 (pleiotropic drug resistance 11)

Cufflinks ID (Cufflinks_ID); total nucleotide length of each predicted transcript (NT_Length); RPKM without salinity stress (RPKM_0) or with salinity stress (RPKM_1); calculated ratio of RPKM (rpkm_1/rpkm_0; Ratio); number of amino acids encoded by putative ORF (AA_Length); and name of similar protein (Description) are listed. Differentially expressed Cufflinks transcripts were identified by the G-test with a 1% false discovery rate. Highly differentially expressed genes (ratio ≥2) derived from different loci that had ORFs predicted by BLASTX search are listed. "-" means a calculated ratio of infinity because the RPKM without salinity stress (RPKM_0) was 0. Detailed data for all unannotated transcripts are listed in Additional file 3: Table S3.

### Comparison of sequence-based and array-based technologies for gene expression profiling

**Figure 6:**
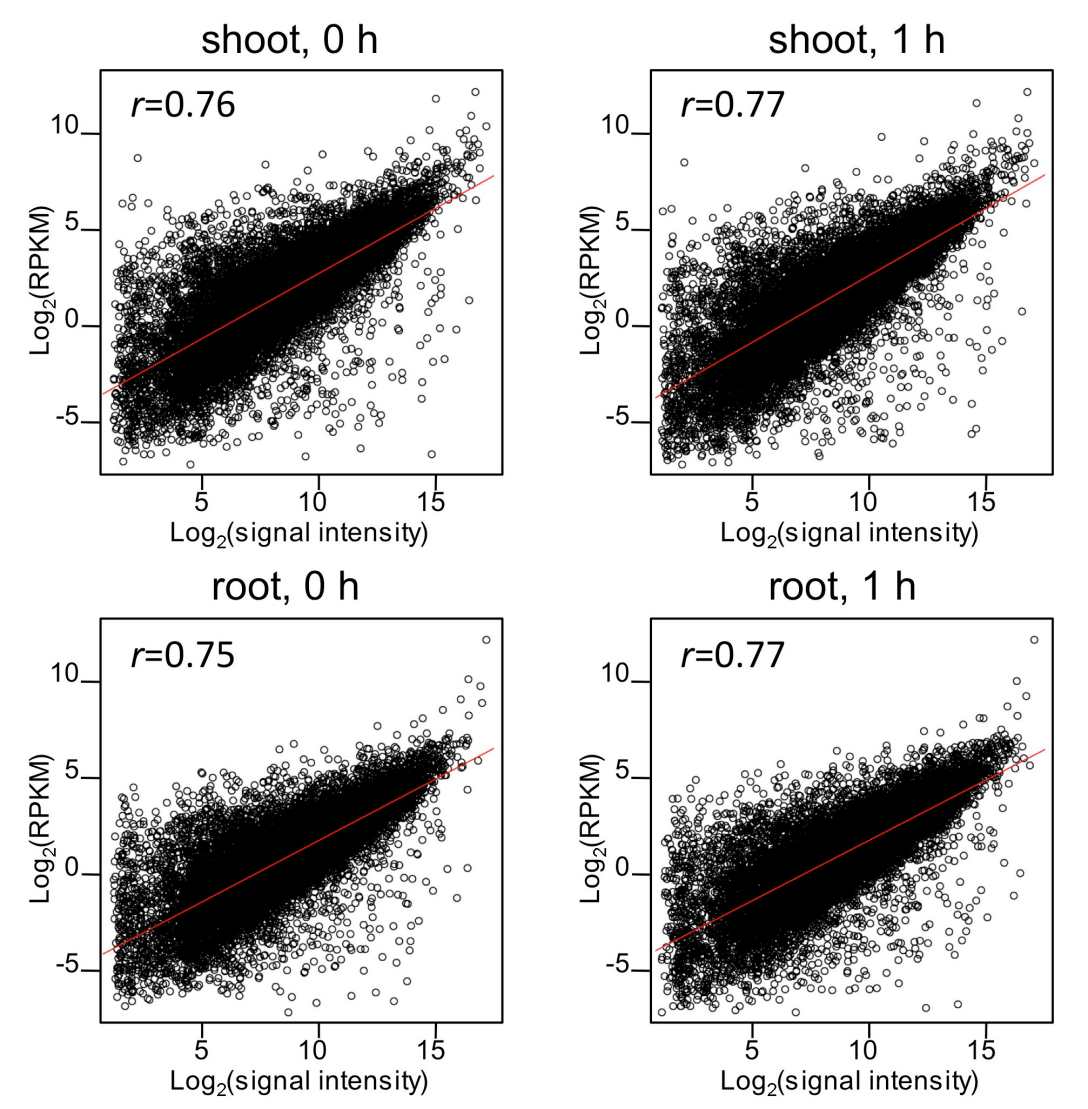
**Comparison of quantification of gene expression by mRNA-Seq and microarray**. For each gene, the normalized intensity (log_2_) from the array is plotted on the horizontal axis, and the corresponding count of RPKM (log_2_) is plotted on the vertical axis. Signal intensity is the average of that of Cy3 and Cy5 (dye-swap experiments). Pearson's correlation coefficient is given in the corner of each plot.

**Figure 7:**
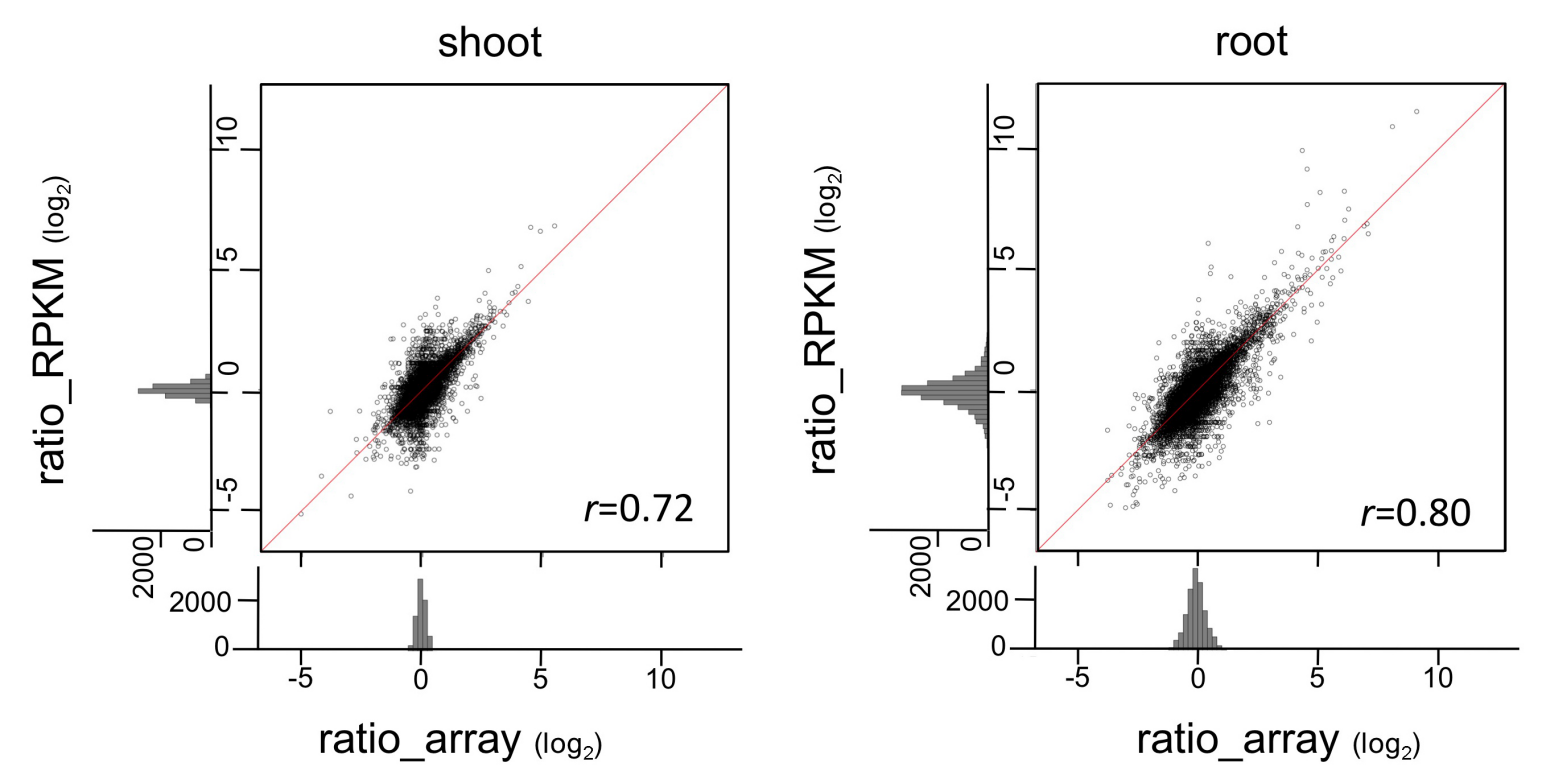
**Comparison of ratios of differential expression calculated by mRNA-Seq and microarray**. For each gene from shoots (*N *= 14,575) and roots (*N *= 14,861), the ratio (log_2_) obtained from the array is plotted on the horizontal axis; the corresponding ratio obtained from RPKM (log_2_) is plotted on the vertical axis. The distributions of the number of transcripts are given outside the plot. Red line indicates where *X *= *Y*. Pearson's correlation coefficient is given in the corner of each plot.

Our sequence-based gene expression profiling was validated against array-based technology. First, signal intensity and RPKM from the same RNA materials were compared. These two independent measures of transcript abundance were correlated (*r *= 0.75-0.77), especially at moderately high signal intensities (Figure 6). However, the correlation was not as strong at extremely high signal intensities (> log_2 _32,768 = 15), suggesting that the array signal intensity was saturated but the RPKM was not (Figure 6, root). Next, the ratios of differentially expressed genes were compared. The ratio obtained from the array and the corresponding ratio obtained from RPKM was highly correlated over a broad range (*r *= 0.72 in shoot and 0.80 in root; Figure 7). The histogram was highest at log_2_1 (= 0), suggesting that most genes were expressed evenly both before and 1 h after salinity stress (Figure 7). However, a few discrepancies were found: increased changes in the expression of 17 genes were found by using the array (> 4×), but not by using mRNA-Seq (< 2×); conversely, increased changes in the expression of 7 genes were found by using mRNA-Seq (> 4×), but not by using the array (< 2×) (Additional file 4: Figure S1). To further examine these discrepancies, we used quantitative real-time polymerase chain reaction (qRT-PCR). The qRT-PCR results suggested that most of the former discrepancy was due to technical inaccuracy in the array experiments. However, qRT-PCR supported only three of the seven mRNA-Seq data in the latter discrepancy (Additional file 4: Figure S1). Despite these discrepancies, our sequence-based approach was generally valid as a gene expression profiling technology for use with previously annotated genes.

## Discussion

### Estimation of variation and abundance of whole transcripts in rice

How many reads are required to cover whole transcripts in the rice cell? As the number of reads increased, the cumulative coverage approached a plateau (Figure 1). We summed four technical replicates (Table 1). RPKM is widely used to calculate the abundance of each transcript and is linear across a dynamic range [16]. The distribution of RPKM of rice genes ranged from 0 to over 10^4 ^(Figure 2); genes involved in photosynthesis in the shoot or in regulation of physiological metals in the root were highly expressed, whereas about 30% of genes had RPKM < 1 (Additional file 2: Table S2). The saturation of sequencing in rice (Figure 1b) was almost the same as in a previous mammalian analysis [16]. According to that analysis, "one transcript in a cell corresponds to 1 to 3 RPKM" [16], so genes having RPKM < 1 might rarely be expressed. However, data on the RNA content of each rice cell are required to calculate the number of existing molecules of RNAs. As rice tissue contains cells of various sizes and types, the relationship between the number of existing molecules and their RPKM has not yet been accurately determined. When we used four technical replicates, about 20% of genes expressed at relatively low levels (RPKM 3-30) did not reach their final RPKM (Figure 1b), suggesting that these model settings were insufficient for calculating the real RPKM of genes expressed at low levels.

Summing of the four technical replicates covered 70.1% of all annotated regions, corresponding to 15.8% of 389 Mb [24] of the rice genome (Figure 1a). This result suggests that these regions were transcriptionally active under the experimental conditions. Even though the cumulative coverage was close to a plateau, the coverage rose gradually; the accumulation of about 95 million reads covered 77.0% of annotated regions (Figure 1a), suggesting that some of the reads expressed at low levels were not sequenced. However, the gradual increase in coverage might have been due to the presence of contaminated genomic DNA or a very small amount of partly processed nuclear RNAs, because intron retention is the most prevalent alternative splicing form in rice [25], as it is in *Arabidopsis thaliana *[26]. Thus, we consider that the summing of four technical replicates of 36-bp reads, corresponding to a total of 1 Gbp of filtered sequences, covered almost all the transcripts in the rice cell under the experimental conditions, although more reads are required to obtain the final RPKM of genes expressed at relatively low levels.

### Identification of unannotated transcripts by mRNA sequencing

mRNA-Seq provides information on whole transcribed genes without the need to rely on annotation (Figure 3), whereas array technology is limited to providing data only on those previously annotated genes and on previously identified ESTs with no known homologies that have corresponding probes on the array. On the basis of the piling up of mapped reads, we predicted 2,795 (shoot) and 3,082 (root) currently unannotated transcripts in RAP-DB (Table 2; Figure 4a). Of the RAP2 unannotated transcripts, 54.6% (1,525/2,795) in shoot and 53.8% (1,659/3,082) in root had not been annotated by Michigan State University (MSU) (data not shown), suggesting that these transcripts were novel transcripts.

Unannotated transcripts included extended parts of previously annotated genes (Figure 4b, c). Extension of 5' exons might contribute to the making of a different start codon or the shifting of the reading frame of previously annotated genes. Extension of 3' UTRs might contribute to microRNA-mediated control of translation or post-transcriptional RNA metabolism [27,28]. For example, mRNA-Seq provided evidence of the existence of extended parts of previously annotated genes and of the differential regulation of their expression. AK240862, previously annotated as a non-protein-coding transcript, had additional predicted exons distal to the 5' end of the previous gene model, and it encoded an indole-3-glycerol phosphate lyase (Additional file 4: Figure S2). Two neighboring genes (AK072595, AK288107) were also similar to the indole-3-glycerol phosphate lyase gene, suggesting that all three genes were tandemly duplicated. Although all three genes were upregulated in response to salinity stress, their tissue specificities and expression levels differed (Additional file 4: Figure S2), suggesting that their functions diversified after gene duplication.

mRNA-Seq also provided evidence of expression of computationally predicted genes. The existence of a number of genes computationally predicted in RAP-DB [15] has not been supported [15] by ESTs [23] or FL-cDNAs [13]. Here, 1,738 (shoot) and 2,297 (root) transcripts identified by mRNA-Seq have been mapped on computationally predicted genes, the presence of which was not supported by experiments, suggesting the validity of the computationally predicted gene models in RAP-DB. We will use these sequence-based transcriptome analyses to improve RAP-DB.

mRNA-Seq provided details of the bridging sequences between exons, suggesting the presence of splicing junctions, whereas array technology--including whole-genome tiling arrays [29]--provides no information on connecting exons. Because reads that bridge exon boundaries are not mapped directly to the genomic sequence, a mapping technique was required. As a first step, the enumeration of all theoretical splicing junctions within annotated transcripts allows the mapping of bridging reads [12,16,30] by using statistical models [31]. We found that 5.0% to 5.7% of reads formed primary bridges with previously annotated exons (Table 1, Unique-bridged); this was not sufficient to discover sequences bridging unannotated transcripts. Programs such as TopHat [10] and G-Mo. R-Se (Gene Modeling using RNA-Seq) [32] are designed to align reads to form potential splice junctions without relying on known splice sites. In this study, sequences flanking potential donor/acceptor splice sites were joined to form canonical (GT-AG) introns between neighboring (but not necessarily adjacent) islands by using TopHat [10]. Even though we used TopHat for our prediction, some of the predicted transcripts remained to be separated--unlike the case with the FL-cDNA sequences--because of the lack of sufficient bridging sequences between the exons (Additional file 4: Figure S3), suggesting that more bridging reads should be sequenced to connect predicted exons. Elongation of the length of each read may also enhance the chance to connect predicted exons.

### Sequence-based transcriptome analysis for capturing salinity stress-inducible genes in rice

mRNA-Seq comprehensively identified salinity stress-inducible genes. Unannotated transcripts had ORFs (Table 2) with a mean length of 123 amino acids (root) or 125 amino acids (shoot) (Figure 5), suggesting that these unannotated transcripts could encode functional proteins. Of the unannotated transcripts, 213 (shoot) and 436 (root) were differentially expressed in response to salinity stress (Table 3, Additional file 3: Table S3). These unannotated transcripts encoded proteins associated with functions such as amino acid metabolism (indole-3-glycerol phosphate lyase) in response to abiotic stress [33], diterpenoid biosynthesis (gibberellin 2-beta-dioxygenase), and mechanosensitive ion channel (MSL2) function [34]. Mechanosensitive ion channels are gated directly by physical stimuli such as osmotic shock and transduce these stimuli into electrical signals [35]. mRNA-Seq also captured previously identified genes involved in salinity tolerance, namely those associated with trehalose synthesis (*OsTPP1*) (Figure 3), dehydrin (*LIP9*), ABA synthesis (*OsABA2*), sugar transport (*OsMST3*), glycerol transferase (*WSI76*), and transcription factors similar to those of the DREB family (Additional file 2: Table S2). A substantial number of transcripts were exclusively upregulated only in the root (Figure 2). As only the root was directly exposed to 1 h of salinity stress, it might take time to induce the expression of more genes in the shoot; *OsTPP1 *(Figure 3) might be expressed in the shoot after 10 h of exposure, as has been found in Yukihikari rice [36]. With these genes, Nipponbare may have the potential to be tolerant to salinity stress.

Rice cultivars such as Nona Bokra and Pokkali are substantially more salinity tolerant than Nipponbare [37], suggesting that the genuine salinity stress tolerance gene might be missing in Nipponbare. The 23 *Oryza *species are geographically, physiologically, and genetically diverse [38], and many of the genes in cultivated rices have been selected by humans under field conditions, not by environmental stress. These essentially missing genes could serve as potential genetic resources for the improvement of cultivated crops. Sequence-based technology can be used to extract such missing genes by the piling-up of short reads on their own genomes without the need to rely on sequence similarity.

### Overcoming the technical inaccuracy

Microarray technology has been used as a sophisticated platform for the expression profiling of previously annotated genes. However, as an array-based technology, evaluation of signal intensities close to background levels tends to cause artifacts in array analysis because of high levels of background noise and/or cross-hybridization [2]; moreover, hybridization efficiency might vary with the probes used, suggesting that the calculation of real molar concentrations is inaccurate. Whereas the Agilent rice 44K Array is designed to quantify 60-mer sequences at the 3'-end of transcripts, mRNA-Seq quantifies transcript abundance on the basis of the number of mapped sequences on the whole gene model. In our study, the two measures of transcript abundance (Figure 6) and change ratios (Figure 7) were highly correlated, as in a previous report [6]. Moreover, for genes expressed at low or extremely high levels (Figure 6, root) and for genes differentially expressed in arrays (Additional file 4: Figure S1a), mRNA-Seq seemed to be accurate. Therefore, mRNA-Seq measures the molar concentrations of genes accurately over a broad dynamic range.

Biological replication is required for identifying differentially expressed genes through statistical analysis, as in array-based analysis. Unfortunately, with sequence-based transcriptome analysis there are greater costs than with microarrays for cDNA preparation and sequencing; this prevented us from performing further experiments. Illumina has improved its sequencing technology. Each read length has been continuously increased. Efficient base calling by using the latest Illumina data analysis pipeline software improved the quality and quantity of reads from the same raw image data. Controlled hydrolysis of RNA before cDNA synthesis substantially improved the uniformity of sequence coverage, as in a previous report [8]. These technical innovations in hardware and software will enable remarkable progress in reducing costs and in increasing the sensitivity of detection of sequences transcribed at low levels, the accuracy of quantification and detection of splice forms, and the prediction of the whole structures of transcripts.

Sequence-based transcriptome analysis has recently been applied to various organisms: *Arabidopsis thaliana *[4,39], yeasts [40,41], *Drosophila melanogaster *[6], and human [5]. During this study, two types of rice transcriptome analysis were reported, focusing on the transcriptional differences in two rice subspecies and their reciprocal hybrids [42] and in eight organs from different developmental stages of *Oryza sativa *L. ssp. *indica *'93-11' [43]. We analyzed salinity stress-inducible transcripts and constructed gene models based on the pilling up of short reads by using the Cufflinks program. This approach should help to discover novel gene models without reliance on gene annotation.

## Conclusions

Microarray-based gene expression profiling is limited to the analysis of annotated genes. In our mRNA-Seq analysis, unannotated salinity stress-inducible transcripts were identified on the basis of the piling up of mapped reads without reliance on gene annotation or FL-cDNA sequences. Some of these novel transcripts had ORFs encoding putative functional proteins and were differentially expressed in response to salinity stress. mRNA-Seq was valid as a gene expression profiling technology for quantifying the abundance of previously annotated genes. Our findings will contribute to improvement of our RAP-DB and to further sequence-based gene expression profiling in various organisms.

## Methods

### Plant material and salt stress treatment

Seeds of rice (*Oryza sativa *L. 'Nipponbare') were germinated in the dark at 28°C on a sterilized germination tray. Germinated seeds were evenly distributed on 96-well PCR plates supported by a plastic container. Seeds were grown in a growth chamber at 28°C, as previously described [44]. After the seedlings had been grown for 7 days, they were transferred on their 96-well plates into containers filled with 150 mM NaCl solution, or with control solution, and placed at 28°C in a growth chamber for 1 h. Four kinds of tissue (normal shoot, normal root, shoot with 1-h salinity stress, or root with 1-h salinity stress) were collected and immediately frozen in liquid nitrogen. For RNA extraction from each treatment group, 10 plants were collected and mixed, to minimize the effect of transcriptome unevenness among plants.

### mRNA sequencing

Total RNA was extracted by using an RNeasy Plant kit (Qiagen, Hilden, Germany). RNA quality was calculated with a Bioanalyzer 2100 algorithm (Agilent Technologies); high-quality (RNA Integrity Number > 8) RNA was used. Total RNA samples (10 μg) were subjected to cDNA construction for Illumina sequencing, in accordance with the protocol for the mRNA-Seq sample preparation kit (Illumina). Oligo(dT) magnetic beads were used to isolate poly(A) RNA from the total RNA samples. The mRNA was fragmented by heating at 94°C for 5 min. First-strand cDNA was synthesized using random hexamer primers for 10 min at 25°C, 50 min at 42°C, and 15 min at 70°C. After the first strand had been synthesized, dNTPs, RNaseH, and DNA polymerase I were added to synthesize second-strand DNA for 2.5 h at 16°C. The ends of double-stranded cDNA were repaired by using T4 DNA polymerase and Klenow DNA polymerase and phosphorylated by using T4 polynucleotide kinase. A single "A" base was added to the cDNA molecules by using Klenow exo-nuclease, and the fragments were ligated to the PE adapters. cDNAs with 200 ± 25-bp fragments were collected. The purified cDNA was amplified by 15 cycles of PCR for 10 s at 98°C, 30 s at 65°C, and 30 s at 72°C using PE1.0 and PE2.0 primers.

### Mapping of short reads, detection of bridging sequences, and prediction of transcripts

For each sample, cDNA was sequenced (single read) by an Illumina Genome Analyzer II. Data on nine technical replicates (nine sequencing lanes of a cDNA sample from root after salinity stress) were accumulated for Figure 1. Data on four technical replicates (four sequencing lanes of each cDNA sample, corresponding to about 27 to 35 million 36-bp reads) were summed for Table 1. In our preliminary experiment, two independent sequencing runs using the same cDNA were highly correlated (*r *= 0.99). The default Illumina pipeline quality filter, which uses a threshold of CHASTITY ≥ 0.6, was used to identify clusters with low signal-to-noise ratios. CHASTITY is defined as "the ratio of the highest of the four (base-type) intensities to the sum of the highest two." Passed filter reads were mapped onto both the Nipponbare reference genome (IRGSP build 4.0) and the spliced exon junction (SEJ) sequences by SOAP ver. 1.11 [45], allowing up to 2 bp of mismatch or up to 3 bp of indels. SEJ sequences were constructed by concatenating the 40 bases at the 3' end of the upstream exon to the 40 bases at the 5' end of the downstream exon for all RAP2 transcripts [14,15] at a locus. To calculate the cumulative coverage of the genome or annotated regions, reads were mapped by BWA (Burrows-Wheeler Aligner) [46] with the default option. To predict transcripts, a series of programs--Bowtie [9], TopHat [10], and Cufflinks [11]--was used. Briefly, mRNA-Seq reads were mapped against the whole reference genome (IRGSP build 4.0) by using Bowtie software. An initial consensus of exon sequences was extracted from the mapped reads. The reads that did not align to the genome but that were mapped to these potential junctions by TopHat were considered to bridge splice junctions. Cufflinks constructs gene models (RPKM ≥ 2, length ≥ 100 bp) on the basis of the exons and bridging sequences predicted by Bowtie and TopHat. ORFs were predicted by BLASTX search against UniProt (Swiss-Prot) and RefSeq (reviewed and validated) or by longest-ORF search (≥20 amino acids).

### Microarray analysis

The same RNA material was shared for use in the Illumina sequencing and the microarray experiments and qRT-PCR analysis. The rice 44K oligo microarray (Agilent Technologies) contained approximately 44,000 60-mer oligonucleotides synthesized on the basis of RAP annotation. For each microarray experiment, 400 ng of total RNAs was used for Cy3- or Cy5-labeled complementary RNA (cRNA) synthesis. DNA microarrays were hybridized for 16 h with 825 ng of Cy3- and Cy5-labeled probes from salinity-stressed or unstressed plants. The microarray experiment was repeated with color-swapping of Cy3 and Cy5. Agilent Feature Extraction Software (ver. 8.5.1.1) was used to quantify microarray images. GeneSpring (ver. 10) software (Agilent Technologies) was used for background subtraction, LOWESS normalization, and extraction of normalized raw signal intensities for all probe sets from each array. Normalized raw signal intensities were compared with the corresponding RPKM. Parts of the signals were removed for further analysis if they were not positive, significant, or above background levels. The hybridization experiments and array scanning were performed at an open laboratory run by the DNA Bank of the National Institute of Agrobiological Sciences (http://www.dna.affrc.go.jp/).

### Quantitative RT-PCR (qRT-PCR)

qRT-PCR primers were designed on the basis of the annotation of the RAP-DB (Additional file 5: Table S4). One microgram of total RNA was reverse-transcribed in a 20-μL reaction mixture of Transcriptor First Strand cDNA Synthesis Kit (Roche, Basel, Switzerland). qRT-PCR was performed in a 20-μL reaction mixture containing 2× SYBR Master Mix (Roche) and 1 μL of cDNA template (1:10 diluted). qRT-PCR of three technical replicates for each sample was performed using a LightCycler480 System with its relative quantification software (ver. 1.2) based on the delta-delta-Ct method (Roche). qRT-PCR was performed for 10 s at 95°C, 5 s at 55°C, and 10 s at 72°C. The detection threshold cycle for each reaction was normalized against the expression level of the ubiquitin gene.

## Authors' contributions

HM, YO, and JW prepared plant materials, performed mRNA extraction and cDNA synthesis; HK, HY, and HI performed sequencing experiments and primary data analysis; YK, HS, HW, and TI performed data analysis; HM, YK, TI and TM designed the study; and HM wrote the manuscript. All authors read and approved the final manusctipt.

## Accession Numbers

All primary sequence read data have been submitted to DDBJ (DNA Data Bank of Japan) [DRA000159], and microarray data have been submitted to the GEO (Gene Expression Omnibus) [GSE20746].

## Supplementary Material

Additional file 1 **Table S1**. Classification of unmapped reads. Click here for file

Additional file 2 **Table S2**. RPKM of RAP2 annotated genes. Click here for file

Additional file 3 **Table S3**. RPKM and ORF predictions of unannotated transcripts. Click here for file

Additional file 4 **Figures S1 to S3**. Figure S1: Discrepancies between mRNA-Seq data and the corresponding array data. The ratio of expression was calculated by array, mRNA-Seq, and qRT-PCR. Vertical lines indicate the ratios of expression of genes after salinity stress. (a) Seventeen genes showed large changes by the array (> 4×), but not by mRNA-Seq (< 2×). Of these, six genes were not differentially expressed by using the G-test with a 1% FDR (asterisks; mRNA-Seq). To further examine these discrepancies, quantitative real-time PCR (qRT-PCR) was used. For qRT-PCR, the averages of three technical replicates are shown. (b) Seven genes showed large changes by mRNA-Seq (> 4×), but not by array (< 2×). These genes were differentially expressed by using the G-test with a 1% FDR (mRNA-Seq). Figure S2: Duplication and differential expression of indole-3-glycerol phosphate lyase genes. Graphs indicate the average depth of reads from mRNA-Seq, as in Figure 3. Gene models in RAP-db based on the full length-cDNA sequences are shown as dark blue boxes. A previously annotated gene, AK240862, had additional exon(s) distal to the 5' end of the previous gene model and encoded an indole-3-glycerol phosphate lyase. Two other neighboring genes (AK072595, AK288107) were also similar to the indole-3-glycerol phosphate lyase gene. Although all three genes were up-regulated in response to salinity stress, their tissue specificities and expression levels were substantially different. Figure S3: Separation of predicted transcripts. Transcripts predicted from shoots (green boxes) or roots (red boxes) by the Cufflinks program are shown. Unlike the annotation in RAP-db based on the full length-cDNA sequence (blue boxes), predicted transcripts are separated because of a lack of bridging sequences between predicted exons. The average depths of reads in the shoot (green graph) and root (red graph) are also shown. Click here for file

Additional file 5 **Table S4**. Primers used in this study. Click here for file
