# Supplementary figures and images for: Massive parallel sequencing of mRNA in identification of unannotated salinity stress-inducible transcripts in rice (Oryza sativa L.)
Source: BMC Genomics. 2010 Dec 2;11:683. doi: 10.1186/1471-2164-11-683 (PMC3016417; doi:10.1186/1471-2164-11-683)

(a)

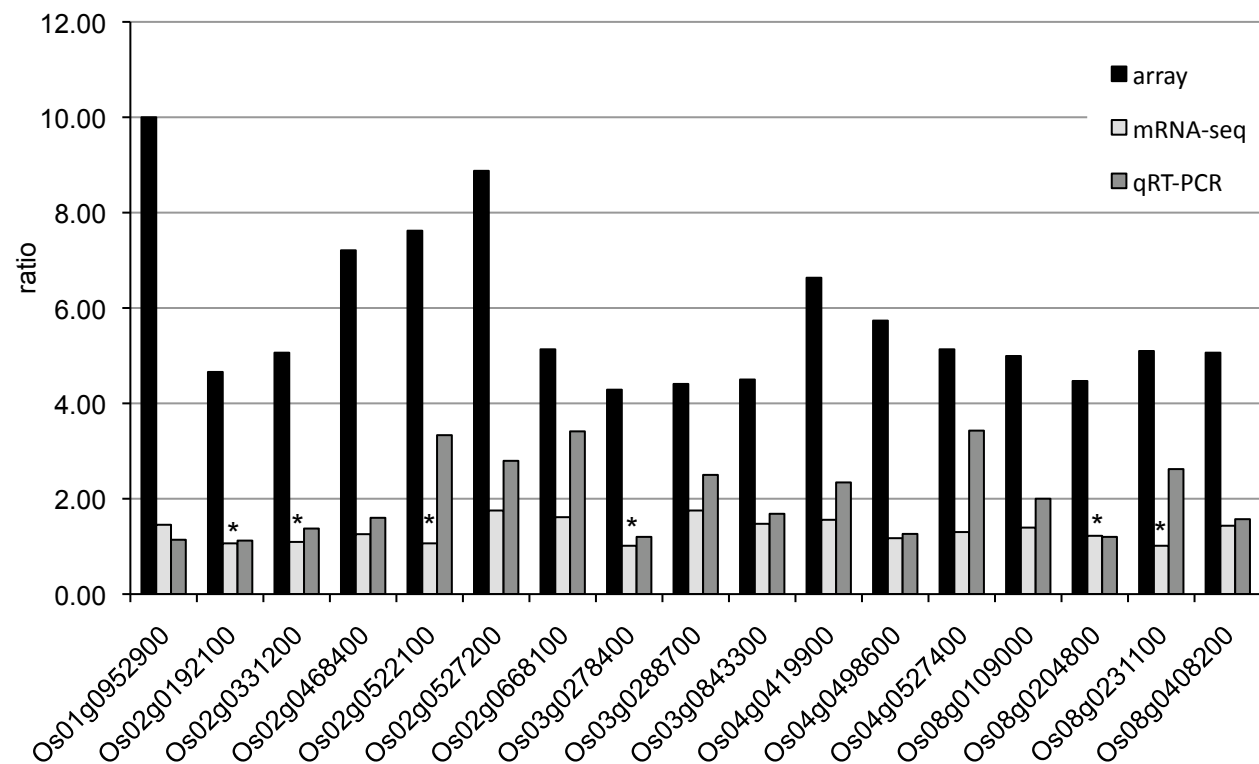

(b)

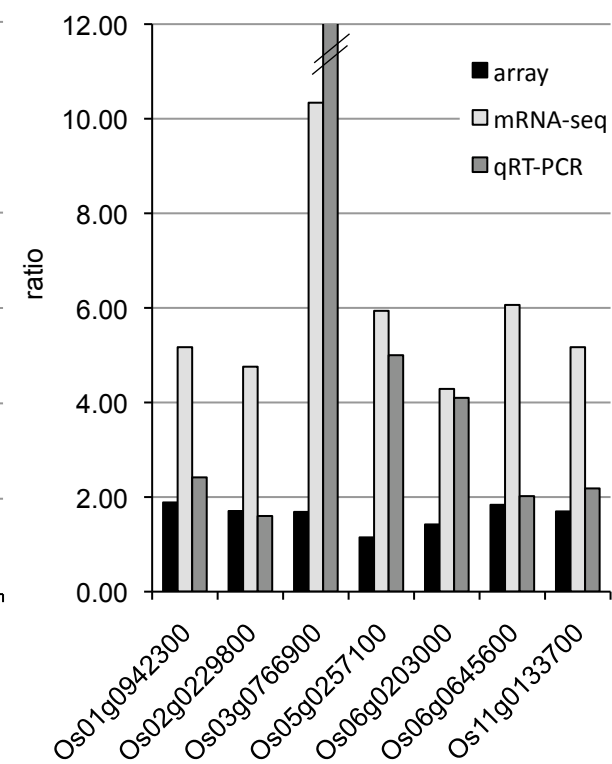

Figure S1

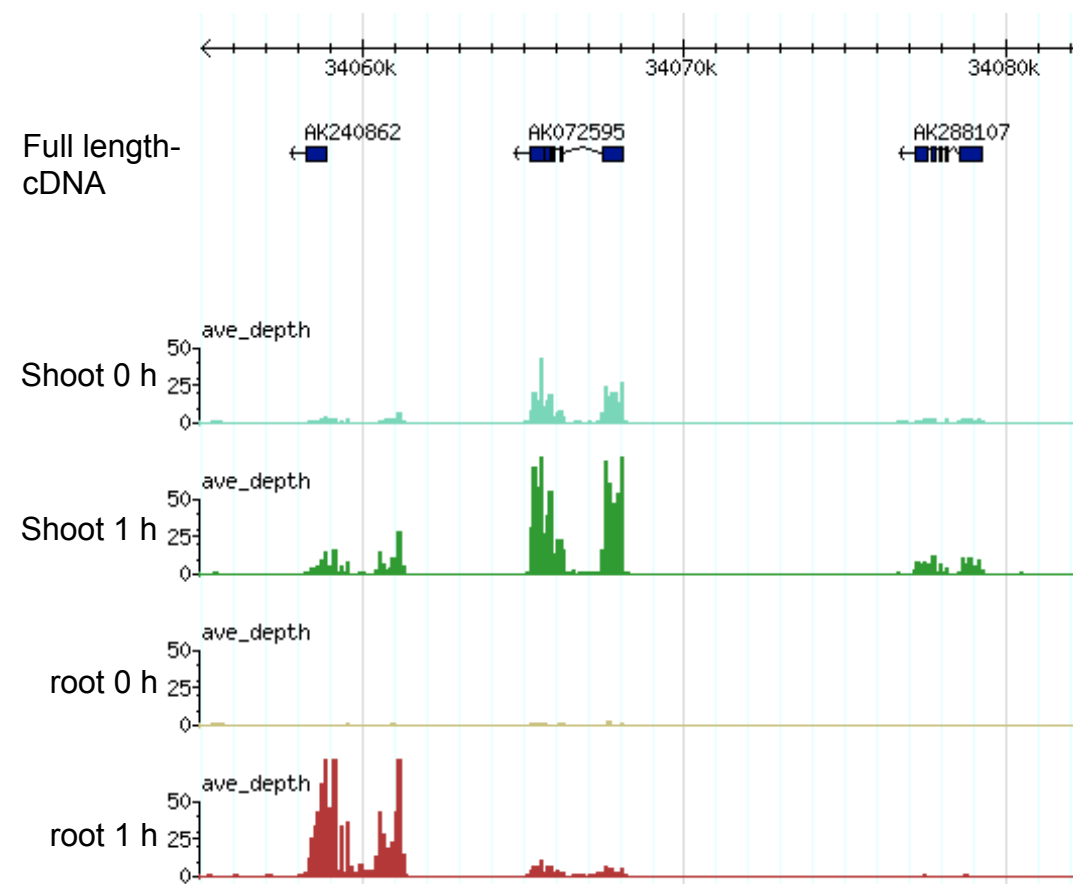

Figure S2

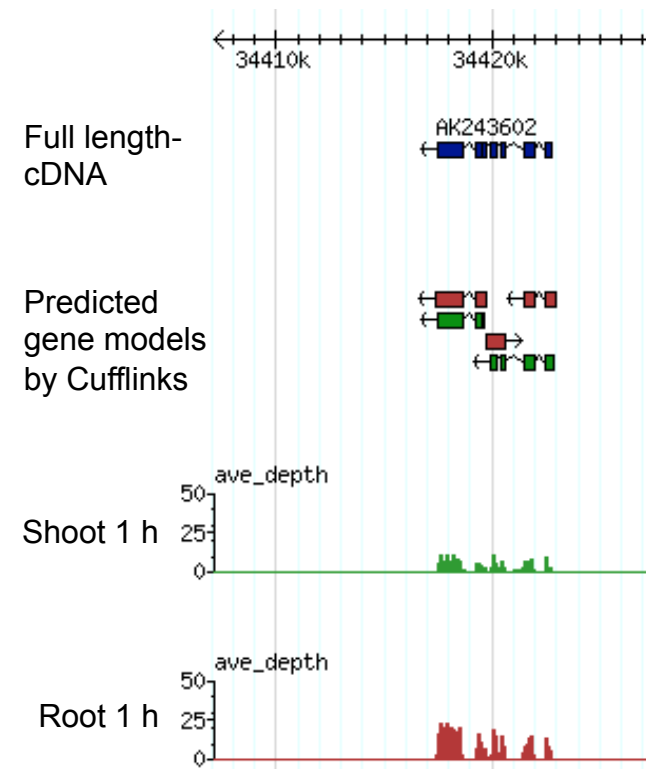

**Figure S3**

Supplement: Additional file 4 — Figures S1 to S3. Figure S1: Discrepancies between mRNA-Seq data and the corresponding array data. The ratio of expression was calculated by array, mRNA-Seq, and qRT-PCR. Vertical lines indicate the ratios of expression of genes after salinity stress. (a) Seventeen genes showed large changes by the array (> 4×), but not by mRNA-Seq (< 2×). Of these, six genes were not differentially expressed by using the G-test with a 1% FDR (asterisks; mRNA-Seq). To further examine these discrepancies, quantitative real-time PCR (qRT-PCR) was used. For qRT-PCR, the averages of three technical replicates are shown. (b) Seven genes showed large changes by mRNA-Seq (> 4×), but not by array (< 2×). These genes were differentially expressed by using the G-test with a 1% FDR (mRNA-Seq). Figure S2: Duplication and differential expression of indole-3-glycerol phosphate lyase genes. Graphs indicate the average depth of reads from mRNA-Seq, as in Figure 3. Gene models in RAP-db based on the full length-cDNA sequences are shown as dark blue boxes. A previously annotated gene, AK240862, had additional exon(s) distal to the 5' end of the previous gene model and encoded an indole-3-glycerol phosphate lyase. Two other neighboring genes (AK072595, AK288107) were also similar to the indole-3-glycerol phosphate lyase gene. Although all three genes were up-regulated in response to salinity stress, their tissue specificities and expression levels were substantially different. Figure S3: Separation of predicted transcripts. Transcripts predicted from shoots (green boxes) or roots (red boxes) by the Cufflinks program are shown. Unlike the annotation in RAP-db based on the full length-cDNA sequence (blue boxes), predicted transcripts are separated because of a lack of bridging sequences between predicted exons. The average depths of reads in the shoot (green graph) and root (red graph) are also shown. [file 1471-2164-11-683-S4.PDF]
